# Expression of Robo4 in the fibrovascular membranes from patients with proliferative diabetic retinopathy and its role in RF/6A and RPE cells

**Published:** 2009-05-29

**Authors:** Lvzhen Huang, Wenzhen Yu, Xiaoxin Li, Yongsheng Xu, Lanjun Niu, Xiangjun He, Jianqiang Dong, Zheng Yan

**Affiliations:** 1 Department of Ophthalmology, Peking University People’s Hospital, Beijing, China; 2 Center Laboratory, Peking University People’s Hospital, Beijing, China

## Abstract

**Purpose:**

Robo4, a member of the roundabout (Robo) family, acts as a neuronal guidance receptor and plays some role in vasculogenesis and angiogenesis. This study investigated the effect of Robo4 on the formation of fibrovascular membranes (FVMs) from patients with proliferative diabetic retinopathy and its roles in choroid–retina endothelial (RF/6A) and human retinal pigment epithelial (RPE) cells.

**Methods:**

RT–PCR and immunohistochemistry were used to determine the levels of mRNA and the presence and distribution of Robo4 in FVMs. Small interfering RNA (siRNA) technology was used to knock down Robo4 expression and to study its effects on RF/6A and RPE cells in vitro. Cell proliferation, migration, spreading, cycling, and apoptosis were assessed with MTT assay, Boyden chamber assay, immunocytochemistry, and flow cytometry. Tube formation by RF/6A on Matrigel was also analyzed.

**Results:**

The level of *Robo4* mRNA was high in FVMs. *Robo4* was expressed in the vessels and fibrous-like tissue co-immunostained for CD31 and GFAP, respectively. *Robo4* siRNA knockdown inhibited cell proliferation and migration. Tube formation by RF/6A cells was also disturbed. Under hypoxic conditions, more apoptotic cells were evident among the knockdown cells than among the control cells (p<0.01).

**Conclusions:**

Robo4 may play a role in the formation of FVMs. Silencing the expression of *Robo4* in RF/6A and RPE cells inhibited their proliferation and reduced their tolerance of hypoxic conditions, suggesting physiologic functions of Robo4 in the cells of the retina.

## Introduction

Retinal angiogenesis and choroid angiogenesis are major causes of vision loss in a variety of clinical conditions, such as retinopathy of prematurity (ROP), age-related macular degeneration (AMD), and diabetic retinopathy [1,2]. Many problems associated with pathological angiogenesis do not arise directly from the growth of new blood vessels but from abnormalities in the newly formed microvasculature, such as increased permeability. Current treatments attempt to abolish these new vessels and/or to prevent their formation. However, a more effective intervention may be to stabilize these abnormal vessels or cause them to mature [3,4].

*Robo4* is a member of the roundabout (*Robo*) family, which contains guidance receptors involved in neurogenesis, which mediate a repulsive signal signal that keeps navigating axons from crossing the midline inappropriately during the process of axonal guidance. The Robo family contains large transmembrane receptors, comprising *Robo1, Robo2, Robo3*, and *Robo4. Robo4* is also known as “magic roundabout” (*MRB*). All known Robo family members have a large extracellular domain composed of five immunoglobulin and three fibronectin motifs, except *Robo4*, the extracellular domain of which consists of only two immunoglobulins and two fibronectin motifs, and is quite different from the other *Robos. Robo4* was first identified as an endothelial-specific member of the roundabout family [5,6] and was later shown by Park et al. [7] to be differentially expressed in activin-like kinase-1^−/−^ mice, which exhibit impaired angiogenesis during development. In silico and in vitro expression analyses indicated that *Robo4* is highly endothelial specific and is strongly upregulated in the vessels of tumors in the brain, colon, and bladder [5,8]. In zebrafish, the knockdown or overexpression of *Robo4* resulted in the temporal and spatial disruption of embryonic vascular development [9], whereas in Robo4-knockout mice, the patterning of the intersomitic and cephalic vessels was normal during early embryogenesis [10], indicating that *Robo4* may have different roles in different species. The expression patterns of *Robo4* differ across species. In zebrafish, *Robo4* is expressed in both neural tissues and the vascular system, whereas in mice, *Robo4* expression is confined to vessels. Murine embryonic *Robo4* expression shows a dynamic pattern within vessels, with expression starting in the larger axial vessels and intersomitic vessels in the earlier embryonic stages, but changing to intersomitic vessel and capillary expression in later stages. However, in the adult, *Robo4* is highly expressed in complex and well organized vascular networks, such as those in the lung, liver, and heart. The expression patterns of *Robo4* observed in embryonic and adult tissues suggest that it may be important in guiding and maintaining the highly reproducible vascular patterning (i.e., the formation of tubular structures) [7]. More recently, defects have been identified in the vascular integrity of *Robo4*-knockout mice, which exacerbate the pathological conditions associated with vascular leakage, suggesting that *Robo4* provides a tonic signal that stabilizes the retinal blood vessels [10]. Robo4 expression has been detected in endothelial cells but not in nonendothelial cell lines, such as fibroblasts and endometrial stoma cells [5,11].

RF/6A, a choroid–retinal endothelial cell line derived from a rhesus macaque fetus, is evolutionarily close to retinal cells derived from humans. It has been identified as of endothelial origin based on cellular morphology and immunodiffusion and is widely used in studies of retinal endothelial cells [12-15].

The retinal pigment epithelium (RPE) has important functions in both the normal eye and under pathological conditions. Recently, vascular endothelial growth factor (VEGF) and its receptor, VEGFR2, have been shown to be highly expressed by the RPE and in the underlying mesenchyme, respectively, at the time of choriocapillary formation in both humans [16] and rodents [17,18].

Although there have been many studies of *Robo4* expression, the biologic function of *Robo4* in the retinal vascular disease is yet to be demonstrated. This study characterizes the expression of *Robo4* in fibrovascular membranes (FVMs) and explores in vitro the role of Robo4 in the retina using two important retinal cell lines, RF/6A and RPE.

## Methods

### Tissue sample

This study protocol was approved by the Ethics Committee of the Peking University, and informed consent was obtained from all patients according to the World Medical Association Declaration of Helsinki. The FVMs specimens were surgically removed from the eyes of 11 patients with type 2 diabetes with proliferative diabetic retinopathy (PDR; 11 eyes) undergoing pars plana vitrectomy with membrane peeling. The patients were six male and five female. Ages ranged between 50 and 75 years (mean age 64.4±11.6). Their duration of diabetes ranged between 6 and 20 years(mean duration 12.2±6.7 years). Six FVM specimens obtained immediately were put into liquid nitrogen were processed for reverse transcription-polymerase chain reaction (RT–PCR) analysis. The remaining five FVM specimens were fixed in a test tube containing 4% paraformaldehyde (PFA) and subsequently embedded in optimum cutting temperature compound (OCT) for immunohistochemistry.

### Cell culture and reagents

RF/6A cells (CRL-1780 cell line) and human RPE cells (D407 cell line) were obtained from the American Tissue Culture Collection (Manassas, VA) and were cultured in Dulbecco’s Modified Eagle Media (DMEM) with 10% fetal bovine serum (FBS, Gibco, Invitrogen, Grand island, NY), 100 units/ml penicillin, 100 µg/ml streptomycin (Sigma, St. Louis, MO) at 37 °C under 5% CO_2_, and 95% humidified air. Before hypoxia, the media was replaced with DMEM free of serum. The cells were then incubated overnight and perfused with 1% O_2_, 94% N_2_, and 5% CO_2_ in a CO_2_ incubator for 24 h [19]. Hiperfect Transfection Reagent was purchased from Qiagen (Hilden, Germany).

### Small interfering RNA and transfection assays

The *Robo4* (GenBank NM019055)-specific siRNAs were chemically synthesized (Table 1). RF/6A and RPE cells were transfected with siRNA by using Hiperfect Transfection Reagent according to the manufacturer’s instructions. Briefly, the original stock of the siRNA was suspended in siRNA suspension buffer (Qiagen, Hilden, Germany, Cat No.301799) provided by the manufacturer. The resulting suspension was aliquoted in the required amounts for each experiment and stored at −20 °C until it was ready to use. On the day of transfection, cells were seeded in plates at the recommended density. For example, 24-well plate: 2–8×10^4^; 96 well plate: 0.5–3×10^4^. The siRNA was then gently introduced into the cells by mixing with the required amount of Hiperfect Transfection Reagent. In our study, the final concentration of siRNA was 10 nm. Nonsilencing siRNA was used to control for any effects of the transfection reagent and siRNA. The assays described here in vitro were performed 48 h post-transfection.

**Table 1:** siRNA sybtype oligonucleotide sequences.

**siRNA subtype**	**Oligonucleotide primers (5′-3′)**
Robo4	F: CCUCAGAGUUCACGGACAUTT
	R: AUGUCCGUGAACUCUGAGGTT
NS	F: UUCUCCGAACGUGUCACGUTT
	R: ACGUGACACGUUCGGAGAATT

### RNA isolation

Total RNA was isolated with Trizol reagent (Invitrogen, Carlsbad, CA) according to the manufacturer’s instructions. After being washed with 75% ethanol, the final RNA extracts were eluted in a 20 µl volume of distilled Diethyl Pyrocarbonate (DEPC)-treated water. The concentration and purity of RNA were measured by spectrophotometer. All the RNA preparations had an OD260:OD280 ratio of 1.9–2.0.

### RT–PCR and Real-time PCR

Two μg of retinal RNAwas converted into cDNA in a total reaction volume of 25 ml, containing 1 mg Oligo (dT) 15, 5 ml M-MLV 5×Reaction Buffer, 1.25 ml dNTPs, 25 units Recombinant RNasin RNase Inhibitor, and 200 Units of M-MLV Reverse Transcriptase. The mixture was incubated for 60 min at 42 °C and reverse transcription was terminated by incubation at 95 °C for 5 min [20]. The single-stranded cDNA was amplified in a polymerase chain reaction(PCR), using sequence-specific primers for glyceraldehydes phosphate dehydrogenase (*GAPDH*) or for RF/6A *Robo4*, or for human *Robo4* (Table 2). The real-time PCR assays were performed according to the manufacturer’s instructions. The real-time PCR assays were performed using IQ Supermix (Bio-Rad, Hercules, CA) with each 20 μl reaction mixture containing 2 µl cDNA, 7.2 µl sterilized water, 10 µl SYBR Green Real-time PCR Master Mix (2×), and 0.8 µl of each primer (10 µM). Amplification was performed in 96-well plates on an iCycler iQ real-time detection system (Bio-Rad). Thermo-cycling conditions consisted of 3 min at 95 °C for activating the iTaq DNA polymerase and 35 cycles of a 20 s, 95 °C denaturation step, a 15 s 63 °C annealing step, and a 15 s 72 °C extension step *Robo4* was normalized to *GAPDH* expression and calculated using the equation: Fold change=2^−ΔΔct^.

**Table 2:** Gene subtype oligonucleotide primers.

**Gene subtype**	**Oligonucleotide primers (5′-3′)**	**Size (bp)**
GAPDH	F: GAGTCCACTGGCGTCTTCAC	120
	R: GTTCACACCCATGACGAACA	
RF/6A Robo4	F: CTGGTTGGAAGACAT GGA	93
	R: ACTTCTCTGGGAAGAGATCC	
human Robo4	F: CCCTGTGCTTGGAACTCAGTG	102
	R: CGCTGATGTACCCATAGGTGG	

### Western blot analysis

Cells were washed with ice-cold phosphate-buffered saline (PBS;, 4 °C, 8.00 g NaCl, 0.20 g KCl, 0.24 g KH_2_PO_4_ and 1.44 g Na_2_HPO_4_ in 1 l distilled water, pH 7.4) for three times every 5 min at room temperature, and prepared using the protein extraction kit and protease inhibitor kit (Pierce, Rockford, IL) and cleared by centrifugation at 12,000x g at 4 °C. The supernatant was collected and the protein content of each lysate was measured using a BCA Protein Assay Kit (Tianlai shengwu jishu, Tianlai, China) according to the manufacturer’s instructions. Equal amounts 20 μg of protein were loaded and analyzed by immunoblotting. Proteins were visualized with enhanced chemiluminescence western blotting detection reagents (Pierce) according to the manufacturer’s recommendations. Band densities of Robo4 proteins were normalized to each β-actin internal control. Western blots were repeated three times and qualitatively similar results were obtained.

### Immunohistochemistry

Membrane tissues were snap-frozen and 6 μm sections were cut. Thawed tissue sections were air dried, placed in 4% paraformaldehyde (PFA) for 20 min for fixing, washed with PBS, and blocked with 10% normal goat serum for 1 h at 37 °C. Next, 1:100 anti-Robo4 polyclonal antibody (Abcam, Cambridge, UK, Cat No.ab10547) with 1:100 anti-GFAP or anti-CD31 (Santa Cruz, Santa Cruz, CA) was applied to the tissue sections at 4 °C overnight and incubated for 1 h at 37 °C with 1:100 fluorescein isothiocyanate (FITC) and tetramethyl rhodamine isothiocyanate (TRITC)-conjugated mouse anti-rabbit and mouse anti-goat secondary antibodies (Santa Cruz), respectively. Following incubation, the slides were washed and cell nuclei were stained with 4’, 6’-diamino-2-phenylindole (DAPI). Images were acquired with a fluorescence microscope equipped with a digital camera. For each of the immunostaining procedures, negative controls included omission of the primary antibody and use of an irrelevant polyclonal or isotype-matched monoclonal primary antibody; in all cases, negative controls showed only faint, insignificant staining.

### Immunocytochemistry

RF/6A and RPE cells (1×10^4^) grown on glass coverslips were washed with PBS three times every 5 min at room temperature and fixed with 4% PFA in PBS and then permeabilized with 0.1% Triton X-100 before they were blocked with 10% goat serum. The slides were incubated with Robo4 antibody at 4 °C overnight and then were washed with PBS and incubated for 1 h at 37 °C with TRITC conjugated mouse anti-rabbit secondary antibody. Following the incubation, slides were washed and cell nuclei were stained with DAPI. Images were acquired with a fluorescence microscope equipped with a digital camera (Leica Microsystems GmbH, Wetzlar, Germany). In each case, preimmune IgG and secondary control incubations were conducted to determine specificity of staining.

### Cytotoxicity assay and cell proliferation assay

Transfection reagent- and siRNA-induced cytotocity was deternmined by a 3-(4,5-dimethylthiazol-2-yl)-2,5-diphenyltetrazolium bromide assay assay (MTT) in accordance with the manufacture’s instruction. In brief, each of the growing cell lines were plated at 1×10^4^ per well in 96 well plates. Three controls were used: one without transfection, one with transfection reagent, and one with control siRNA. After incubating 48 h, MTT was added and the cells were incubated for a further 4 h. Formazan crystals that formed were then dissolved by the addition of dimethyl sulfoxide (DMSO; 100 μl/well). Absorbance at 570 nm was measured using an ELISA plate reader (Dynatech Medica, Guernsy, UK) [21]. Cell proliferation was measured by a modified MTT assay on 24, 48, 72, 96, and 120 h. Media were changed on day 3. Each experiment was undertaken using three wells and was performed at least three times.

### Cell attachment assay

Ninety-six-well plates coated with 1.25 μg/ml fibronectin in 100 μl of PBS were put into the incubator overnight at 4 °C. Transfected cells (1×10^4^) were trypsinized, added to each well, and allowed to attach for 6 h [22]. The cells were then washed gently twice with PBS, and 150 μl fresh medium was added to each well with MTT. The absorbance was measured with an ELISA plate reader at 570 nm. We used three different wells to detect the cell attachment and repeated all the experiments three times.

### Cell spreading assay

After transfection, 1×10^4^ cells were trypsinized and added to fibronectin-coated coverslips in DMEM and 10% serum at 37 °C for 2 h. After they were washed with PBS three times, cells were fixed with 4% PFA in PBS, and then were stained with vimentin and DAPI. Cell spreading was characterized by the formation of a clearly defined cytoplasm halo around the cell nucleus, and spreading was quantified by analysis of images of four separate microscope fields. (Leica Microsystems GmbH, Wetzlar, Germany). Quantitation was performed by measuring the ratio of cytoplasm area to nucleus area of cells in each field using Image-Pro Plus software (Media Cybernetics Ins, Bethesda, MD). We used three wells to detect cell spreading and repeated all the experiments three times.

### Cell migration

Migration assay was performed as described before [23]. Briefly, 2×10^4^ cells were placed in the upper chamber in a final volume of 200 μl of serum-free medium. Next 10% FBS was placed in the bottom chamber for a final volume of 600 μl. All migration assays were conducted for 4 h at 37 °C. At the end of the assay, the cells were fixed in 4% PFA and stained with DAPI for 15 min. Remaining cells were wiped away with a cotton bud, and the membrane was imaged. The number of cells from five random fields of view was counted.

### Tube formation

The tube formation assay was conducted to investigate the effect of *Robo4* siRNA on RF/6A in vitro. Aliquots (150 μl) of matrigel solution were poured into the 48 well plates (repeated 2 more times), and the plates were incubated at 37 °C for 1 h in a 5% CO_2_ incubator to form a matrigel gel [21]. RF/6A cells (1×10^4^ per well) treated with siRNA for 48 h were seeded on the matrigel and cultured in DMEM medium. The networks in matrigel from five randomly chosen fields were counted and photographed under a microscope.

### Flow cytometry

Each of the cell lines (1×10^6^) were seeded in 6-well plates and treated with NS siRNA or *Robo4* siRNA transfection reagent at normoxia or hypoxia for 48 h. Then dividend into the following groups: *NS* group at normoxia; *Robo4* siRNA treated at normoxia; *NS* group with hypoxia; and *Robo4* siRNA with hypoxia. Cells were detached using EDTA, washed in cold PBS (4 °C), and stained with propidium iodide and Annexin-V-FITC (Becton Dickinson, Franklin Lakes, NJ) for 15 min at room temperature in the dark. For cell cycle analysis, cells were treated with the BD Cycletest™ Plus DNA Reagent Kit (Becton Dickinson) according to the manufacturer’s instructions. Samples were analyzed using a FACS Caliber cytometer (Becton Dickinson, Franklin Lakes, NJ). We used three samples in one experiment and repeated it.

### Statistical evaluation

All data were presented as mean±SD and evaluated for normality of distribution. Statistical differences were evaluated using ANOVA followed by Student–Newman–Keuls test for multiple comparisons and the Student’s *t*-test for pairwise comparisons. p<0.05 was considered significant.

## Results

### Expression of *Robo4* mRNA in FVMs

**Figure 1:**
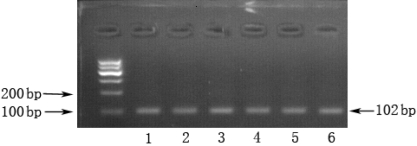
RT–PCR analysis of *Robo4* in FVMs derived from proliferative diabetic retinopathy patients. After 35 cycles, 10 μl each sample was separated electrophoretically through a 1.5% Tris–acetate–EDTA agarose gel, and an expected product at 102 bp for *Robo4* were stained with ethidium bromide. 1–6 was on behalf of 6 patients.

RT–PCR was performed to verify the expression of *Robo4* in the FVMs. As shown in Figure 1, the expected 102 bp PCR product, representative of *Robo4*, was detectable on 1.5% agarose gels in six (100%) of the six samples of tissues.

### Immunohistochemical detection of Robo4 in FVMs

**Figure 2:**
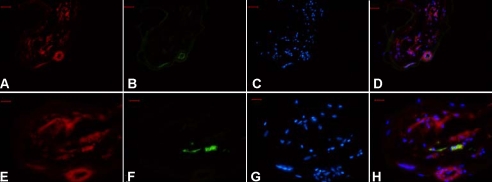
Robo4 expression in FVMs from a 60-year-old patient with a 10 year history of diabetes. Immunofluorescent staining showed Robo4-positive (**A, E**), CD31-positive (**B**), and GFAP-positive (**F**) staining in FVMs. Cell nuclei were stained with DAPI (**C**,**G**). The colocalization of Robo4 and CD31 (**D**), and Robo4 and GFAP (**H**) are also shown. Bar graph denote 50 μm.

To investigate the presence and distribution of Robo4 in FVMs, we stained the sections with an anti-Robo4 antibody (Figure 2A), an anti-CD31 antibody (an endothelial cell marker; Figure 2B), and an anti-GFAP antibody (a glial cell marker; Figure 2F). Consistent with previous results on the staining patterns of tumors [5,8], the Robo4 antibody was expressed in the vessels of the FVMs. We found that Robo4 was coexpressed with GFAP, a marker of glial cells, unlike previous studies in which Robo4 was not expressed in the neural tissues of mammals [5,7].

### *Robo4* siRNA specifically knocks down *Robo4* RNA and protein

**Figure 3:**
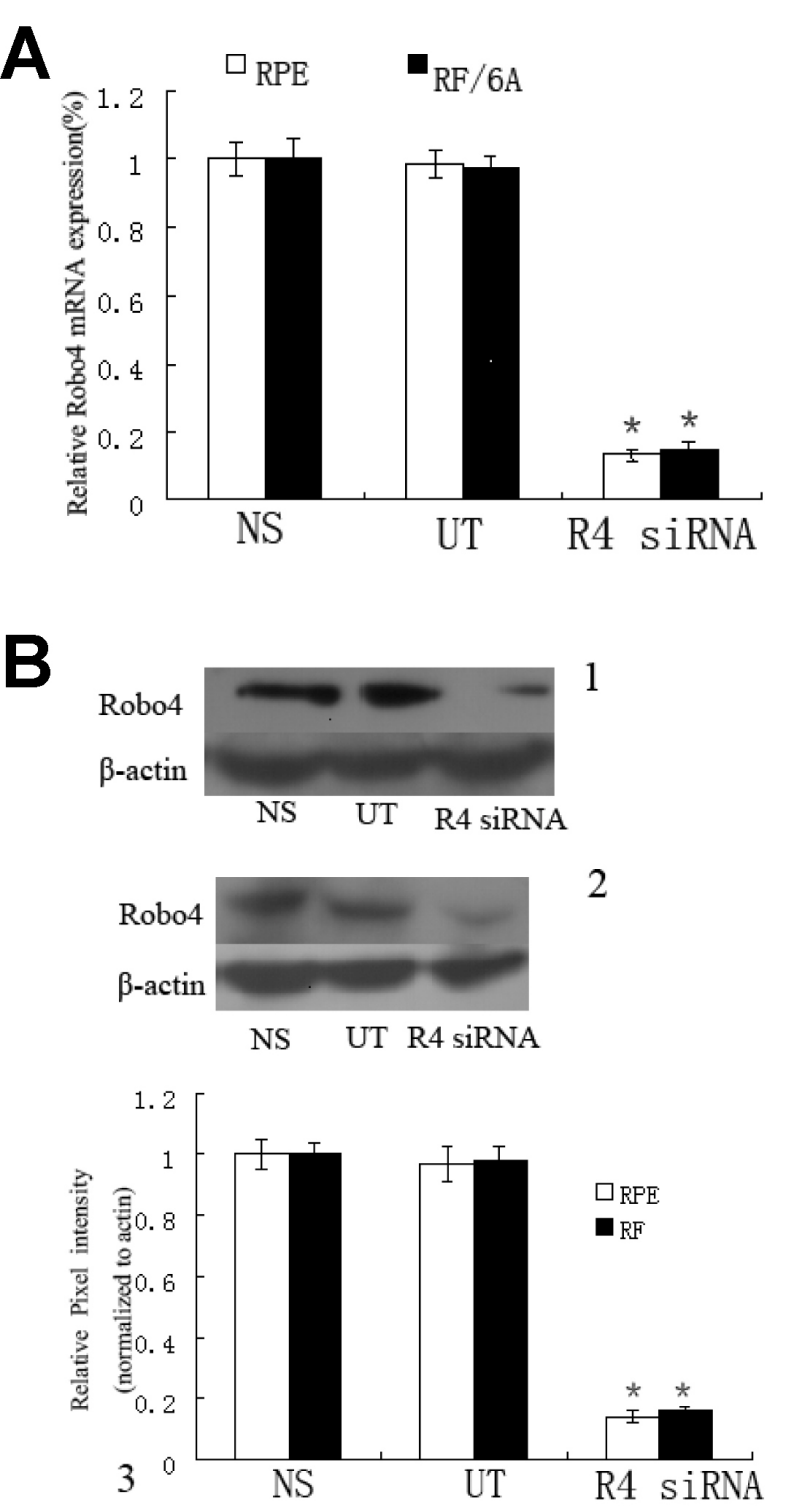
*Robo4* siRNA specifically knocks down Robo4 mRNA and protein level. **A:** *Robo4* expression in RF/6A and human RPE cells was significantly knocked down at the mRNA level, measured by real-time RT–PCR 48 h after transfection. **B:** The protein expression of Robo4 protein in RF/6A and human RPE cells was measured by immunoblotting, normalized to β-actin expression in RF/6A and RPE cells. One was a representative photograph of the western-blot analysis of *Robo4* expression in RF/6A; 2 was a representative photograph of the western-blot analysis of *Robo4* expression in RPE; 3 was the data of the relative Robo4 protein in the NS, UT and *Robo4* siRNA-treated cells. Values are the means±SD of at least three independent experiments. Abbreviations: control siRNA treated cells (NS); untransfected cells (UT); *Robo4* siRNA-treated cells (*Robo4* siRNA). Asterisks denote values signigicantly different from *Robo4* siRNA-treated group compared to NS and UT group (p<0.01). The pixel intensity of NS was set to 100%.

**Figure 4:**
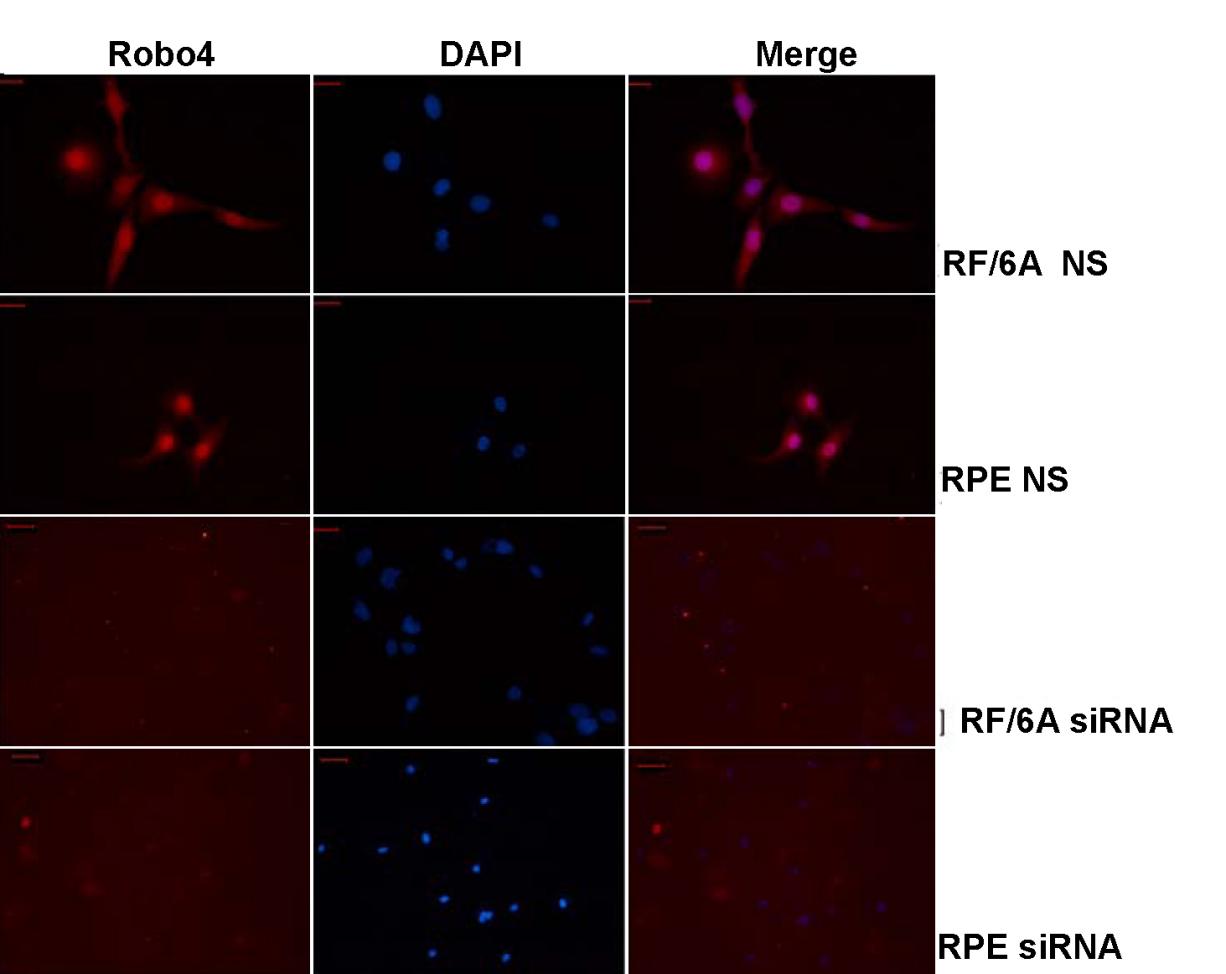
Immunocytochemical assays for Robo4 in RF/6A cells and human RPE cells. The fluorescence, representing the expression of Robo4 in both cell lines, was very strong in NS siRNA-treated cells but was barely detectable in the *Robo4*-siRNA-treated cells. Abbreviations: control siRNA treated cells (NS); *Robo4* siRNA-treated RF/6A cells (RF/6A siRNA); *Robo4* siRNA-treated RPE cells (RPE siRNA). Bar denote 50 μm.

We first determined the expression of Robo4 in RF/6A and RPE cells (Figure 3, Figure 4). Robo4 expression in RF/6A and RPE cells was significantly knocked down at both the mRNA and protein levels, determined by real-time RT–PCR and western blot assays, respectively. At the mRNA level, real-time RT–PCR demonstrated that specific siRNA depleted *Robo4* mRNA levels by 85% and 87% in RF/6A and RPE cells, respectively, consistent with the strong depletion of Robo4 protein expression (p<0.01; Figure 3A,B). In contrast, there was no significant difference between the cells transfected with control siRNA (NS) and the untransfected (UT) cells (p>0.05). Immunocytochemical imaging of the expression of Robo4 in RF/6A and RPE cells confirmed the knockdown of Robo4 (Figure 4). These results suggest that we have generated specific knockdown reagents that can selectively target Robo4 in both RF/6A and RPE cells.

### Transfection of *Robo4*-specific siRNA is not cytotoxic for RF/6A or RPE cells

**Figure 5:**
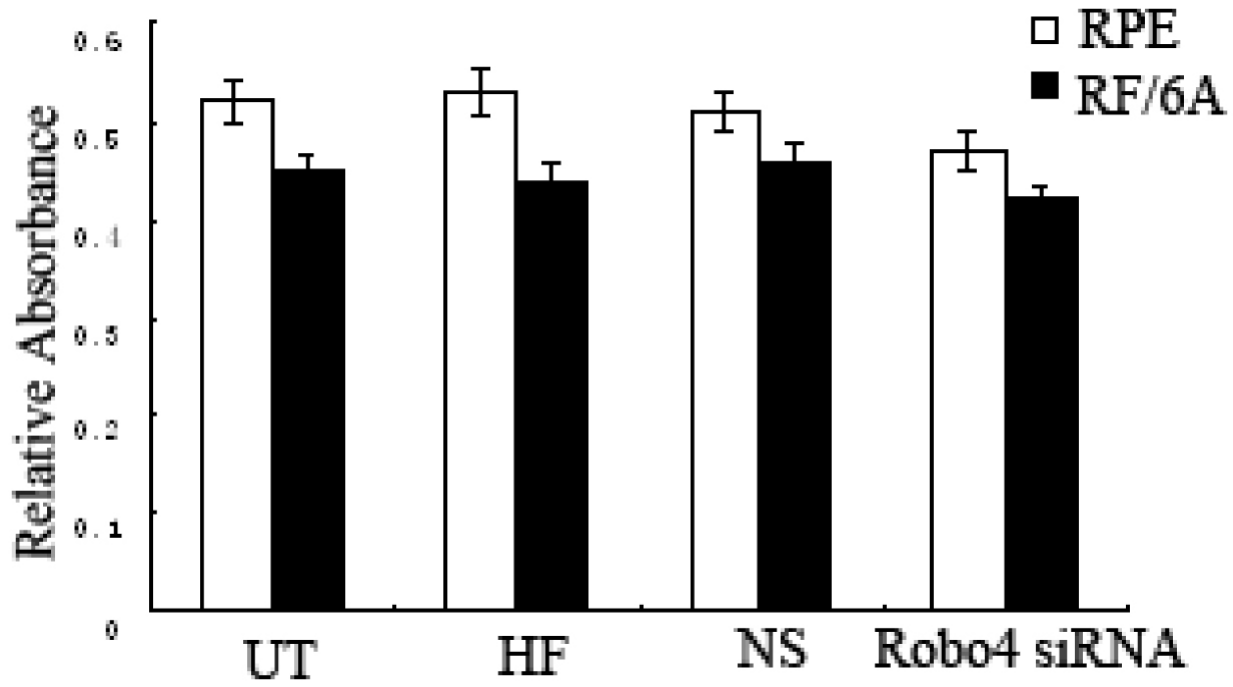
Cytotoxicity caused by the transfection of RF/6A and human RPE cells. Both cell lines were transfected with control siRNA or HiPerFect Transfection Reagent and incubated for 48 h. The inhibition of cellular viability was measured with an MTT assay. Values are the means±SD of at least three independent experiments. Abbreviations: untransfected cells (UT); cells treated with HiPerfect Tranfection Reagent (HF); control siRNA-treated cells (NS); *Robo4* siRNA-treated cells (*Robo4* siRNA).

We performed an MTT assay to detect any transfection-induced cytotoxicity. The mitochondrial dehydrogenases of viable cells convert MTT to purple formazan crystals after incubation for 4 h. The absorbance of the solubilized formazan product correlates with the total metabolic activity of the living cells. There was no significant difference in the cell viability of the UT, HF (cells treated with HiPerFect Tranfection Reagent only), and NS groups (p>0.05; Figure 5).

### *Robo4* regulates cell attachment, proliferation, spreading, and migration

**Figure 6:**
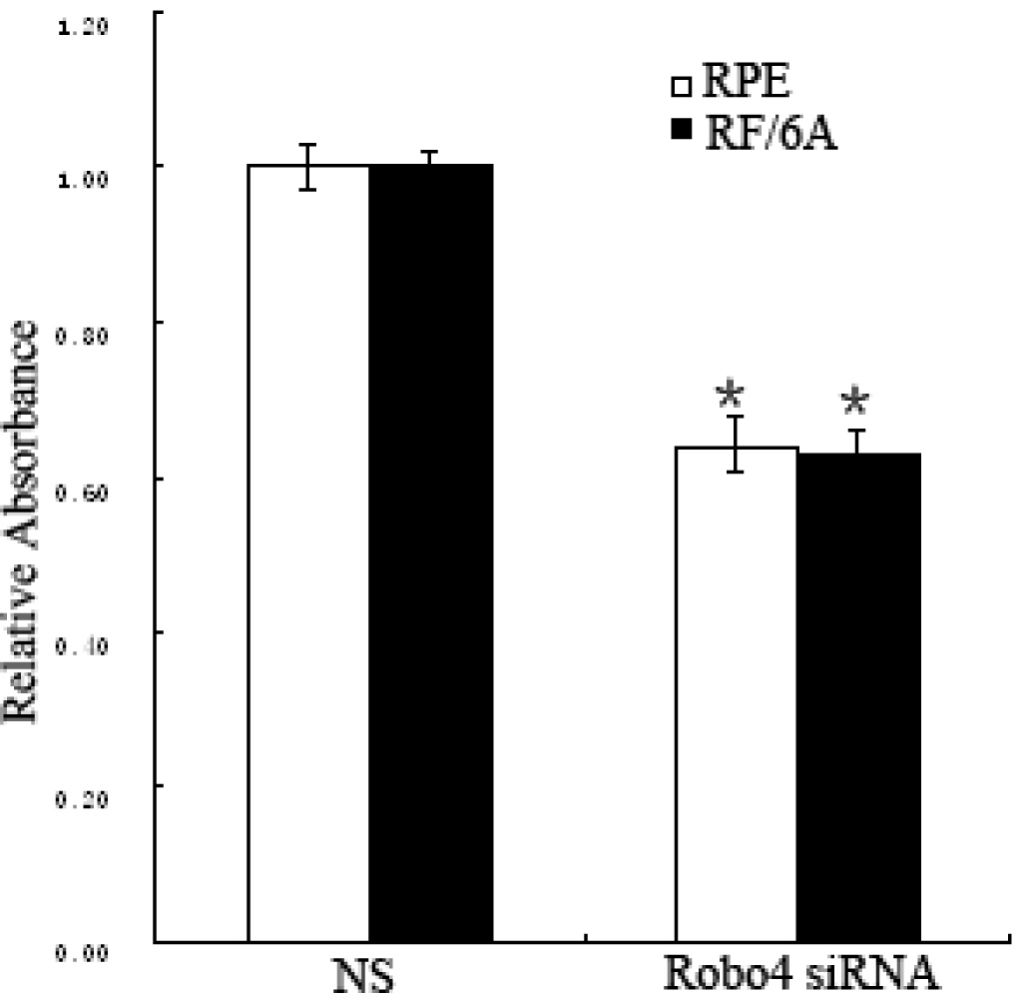
Effects of Robo4 on the attachment of RF/6A and RPE cells. Cell attachment was assessed after 6 h incubation and subsequent MTT assay. Values are the means±SD of at least three independent experiments. Asterisks denote values significantly different from those of cells treated with *Robo4* siRNA compared to NS siRNA (p<0.01). Abbreviations: control siRNA-treated cells (NS); Robo4 siRNA-treated cells (R4 siRNA). The absorbance of NS was set to 100%.

**Figure 7:**
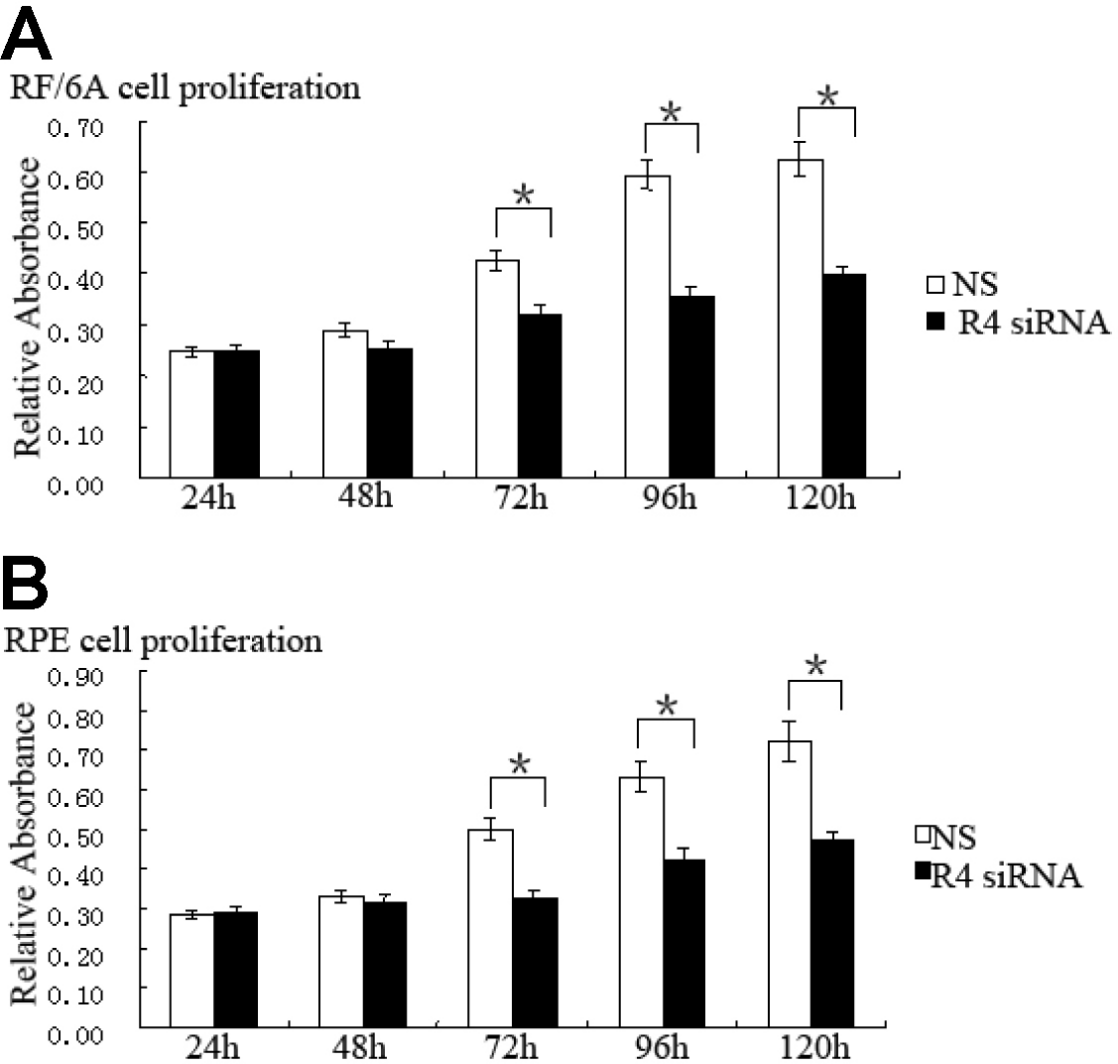
Effect of Robo4 on the proliferation of RF/6A and human RPE cells. RF/6A (**A**) and RPE (**B**) cell proliferation was measured with an MTT assay at 24 h, 48 h, 72 h, 96 h, and 120 h. Values are the means±SD of at least three independent experiments. Asterisks denote values significantly different from those of cells treated with *Robo4* siRNA compared to NS siRNA (p<0.01). Abbreviations: control siRNA-treated cells (NS); Robo4 siRNA-treated cells (R4 siRNA).

In the cell attachment assay, the *Robo4* siRNA treatment reduced the attachment capacity of RF/6A cells by 36% (p<0.05) and of RPE by 35% (p<0.01; Figure 6) after 6 h compared with that of the NS group. The NS and UT groups were not significantly different in their capacities for cell attachment (p>0.05; data not shown). The suppression of Robo4 reduced cell proliferation by 25% in RF/6A cells at 72 h compared with the proliferation of the NS cells (p<0.01), and the suppression peaked at 40% on the fourth day (p<0.01). The suppression of *Robo4* in RPE cells reduced cell proliferation by 35% at 72 h compared with that of the NS cells (p<0.01; Figure 7).

**Figure 8:**
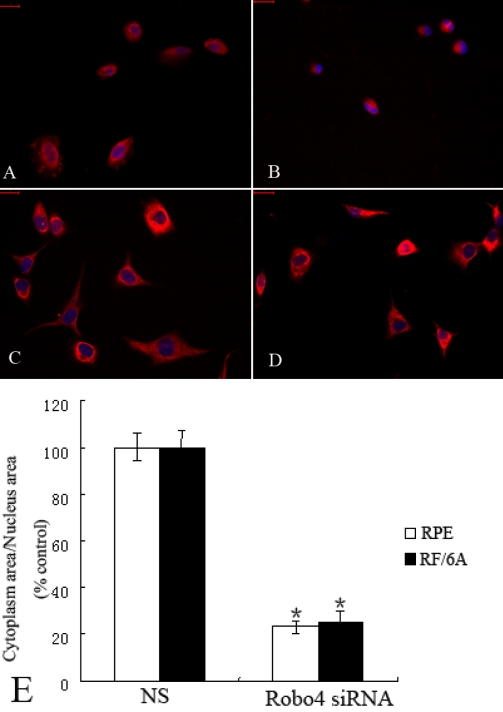
Effects of Robo4 on the spreading of RF/6A and RPE cells. Cell spreading was quantified by measuring the ratio of the cytoplasm area to the nuclear area in cells in each field. **A:** Cell spreading of NS siRNA-treated RPE cells. **B**: Cell spreading of *Robo4* siRNA-treated RPE cells. **C**: Cell spreading of NS siRNA-treated RF/6A cells. **D**: Cell spreading of *Robo4* siRNA-treated RF/6A cells. **E:** The data of relative ratio of the cytoplasm area to the nuclear area in NS and *Robo4* siRNA group cells. Values are the means±SD of at least three independent experiments. Asterisks denote values significantly different from those of cells treated with *Robo4* siRNA compared to NS siRNA (**E**, p<0.01). Abbreviations: control siRNA-treated cells (NS); *Robo4* siRNA-treated cells (R4 siRNA). The ratio of the NS group was set to 100%. Bar denote 100 μm.

In the cell spreading assay, Robo4-knockdown RF/6A and RPE cells spread less well than the control cells. As shown in Figure 8, the ratios of the cytoplasm area to nuclear area in the RF/6A and RPE cells treated with *Robo4* siRNA and the NS group were 23% and 25%, respectively, of the ratio of the NS group (p<0.01; Figure 8).

**Figure 9:**
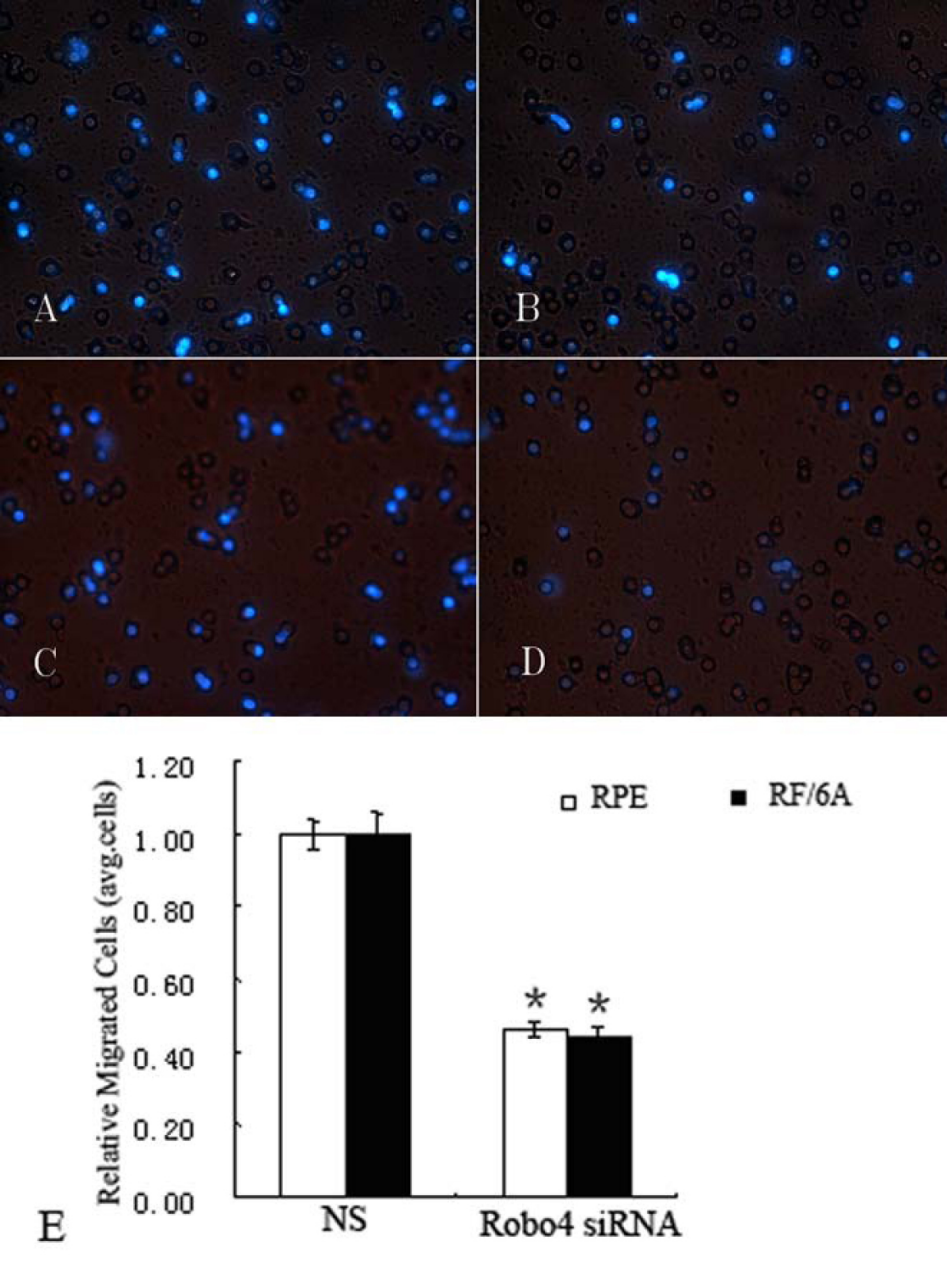
Effect of Robo4 on the migration of RF/6A and human RPE cells. The migratory activity of both cell lines was estimated based on the number of cells that had migrated through the filter of the chamber. **A:** Migrated cells of NS siRNA-treated RF/6A cells. **B:** Migrated cells of *Robo4* siRNA-treated RF/6A cells. **C:** Migrated cells of NS siRNA-treated RPE cells. **D:** Migrated cells of *Robo4* siRNA-treated RPE cells. **E:** The data of relative migrated cells in NS and *Robo4* siRNA group cells. Values are the means±SD of at least three independent experiments. The results showed that the number of migrating cells in the *Robo4* siRNA-treated group was less than in the control siRNA-treated group (**E**, p<0.01). Abbreviations: control siRNA treated cells (NS); *Robo4* siRNA-treated RF/6A cells (RF/6A siRNA); *Robo4* siRNA-treated RPE cells (RPE siRNA). The migrated cells of NS group was set to 100%.

Next, we explored the role of Robo4 in the migration of RF/6A and RPE cells using a modified Boyden chamber in which the RF/6A and RPE cells migrated through a porous membrane. As shown in Figure 9, the mean numbers of migrated cells among the *Robo4*-siRNA-treated RF/6A and RPE cells were significantly lower than the number of migrated control cells (p<0.05). In contrast, the mean numbers of migrated cells in the NS and UT groups did not differ significantly (p>0.05, data not shown).

### Suppressing Robo4 disturbs tube formation by RF/6A cells

**Figure 10:**
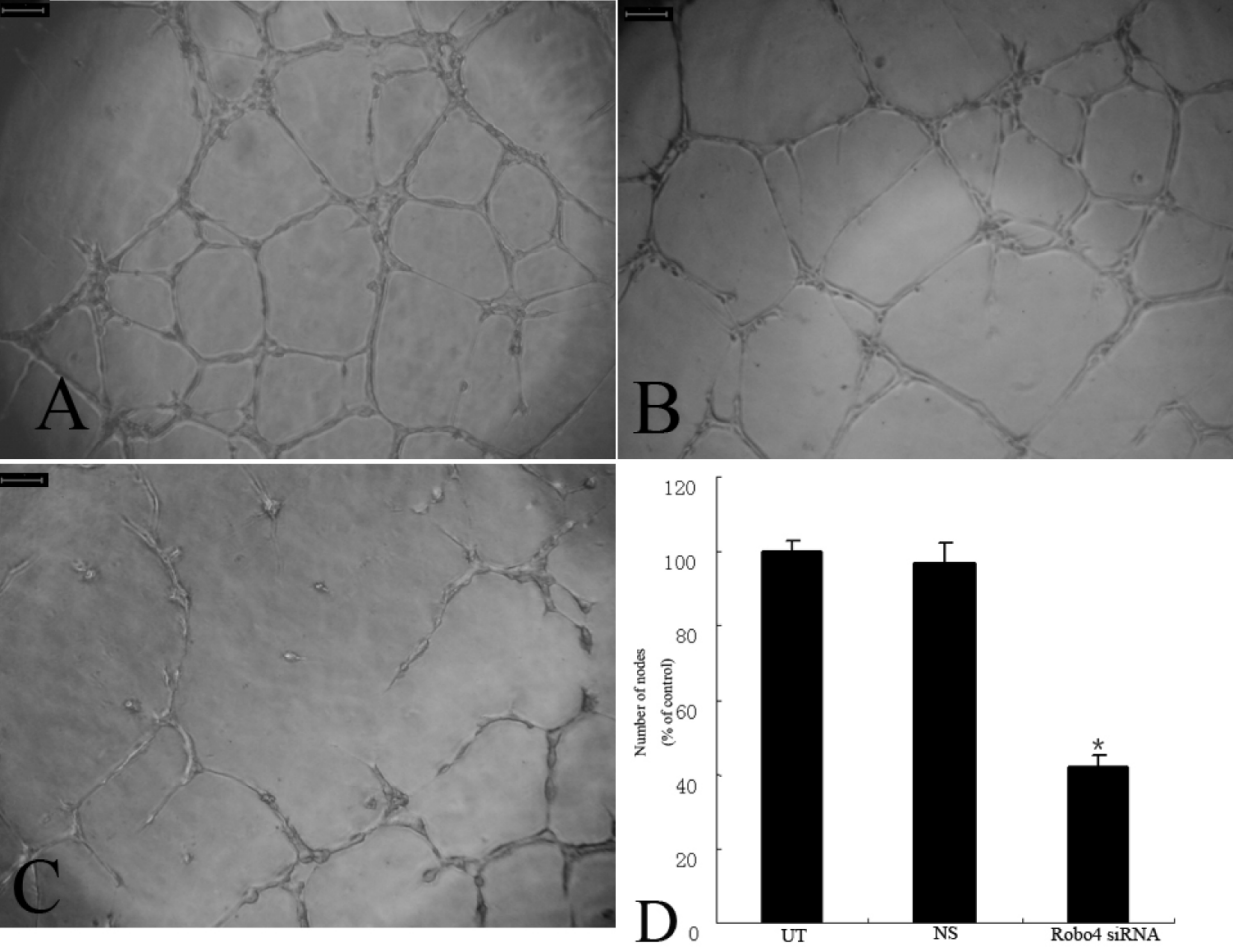
Effect of Robo4 on the tube formation of RF/6A cells. Untransfected cells (**A**), NS siRNA-treated cells (**B**) and *Robo4* siRNA-treated cells (**C**) were plated on Matrigel as described in Methods. After 24 h of incubation, UT and NS group cells formed well organized capillary-like structures (**A, B**), while *Robo4* siRNA group cells ability to organize was severely compromised (**C**). Values are the means±SD of at least three independent experiments. Asterisks denote values significantly different from those of cells treated with *Robo4* siRNA compared to NS siRNA and UT group (**D**, p<0.01). Abbreviations: untransfected cells (UT); control siRNA-treated cells (NS); *Robo4* siRNA-treated cells (Robo4 siRNA) . The number of UT group was set to 100%.

In a Matrigel assay, Robo4-knockdown RF/6A cells showed an impaired capacity to form a regular network: fewer branches were formed and the network was uneven (p<0.05; Figure 10). There was no significant difference between the NS and UT groups (data not shown).

### Cell cycle and cell apoptosis

**Figure 11:**
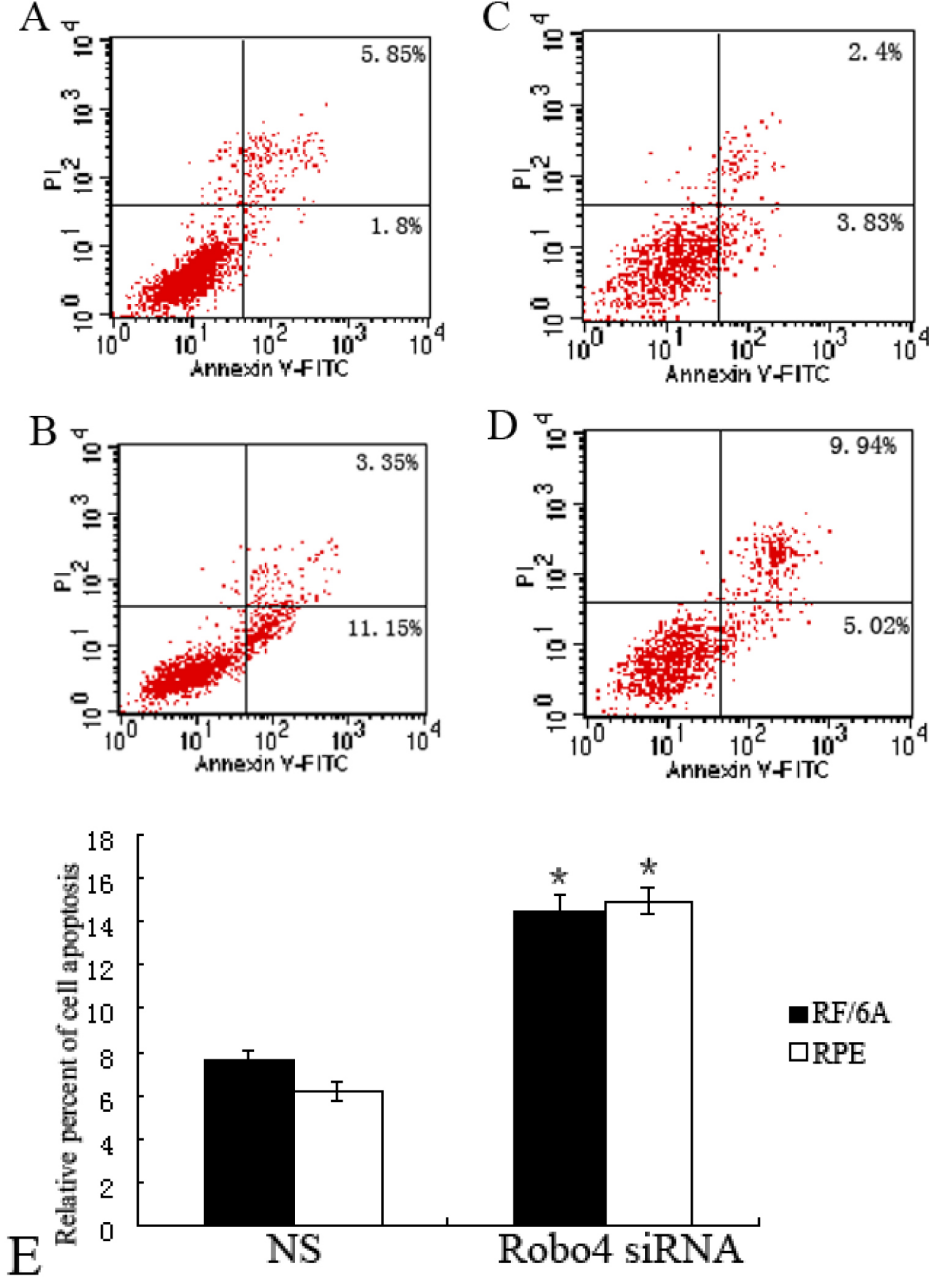
Effect of Robo4 on the apoptosis of RF/6A and RPE cells. **A:** Cell apoptosis of NS siRNA-treated RF/6A cells. **B:** Cell apoptosis of *Robo4* siRNA-treated RF/6A cells. **C:** Cell apoptosis of NS siRNA-treated RPE cells. **D:** Cell apoptosis of *Robo4* siRNA-treated RPE cells. **E:** The data of relative cell apoptosis in NS and *Robo4* siRNA group cells. The normal living cells (bottom left quadrants) showed low annexin V and propidium iodide staining. The early apoptotic cells (bottom right quadrants) showed high annexin V staining but low propidium iodide staining. The late apoptotic cells (top right quadrants) showed high annexin V and propidium iodide staining. The percentages of cells in the quadrants are indicated within the quadrant of all panels. Representative results of three separate experiments are shown. Values are the means±SD of at least three independent experiments. Asterisks denote values signigicantly different from those of cells treated with Robo4 siRNA compared to NS siRNA and UT group (**E**, p<0.01).

As shown in Figure 11, in both cell lines, *Robo4*-siRNA-treated cells exhibited more cell apoptosis than did the NS cells under hypoxic conditions (p<0.01), whereas under normoxic conditions, there was no significant difference in apoptosis between the siRNA-treated and NS groups (data not shown). These results suggest that *Robo4*-siRNA-treated cells have a reduced tolerance for hypoxia, whereas *Robo4* siRNA did not increase cell apoptosis under normal conditions.

**Figure 12:**
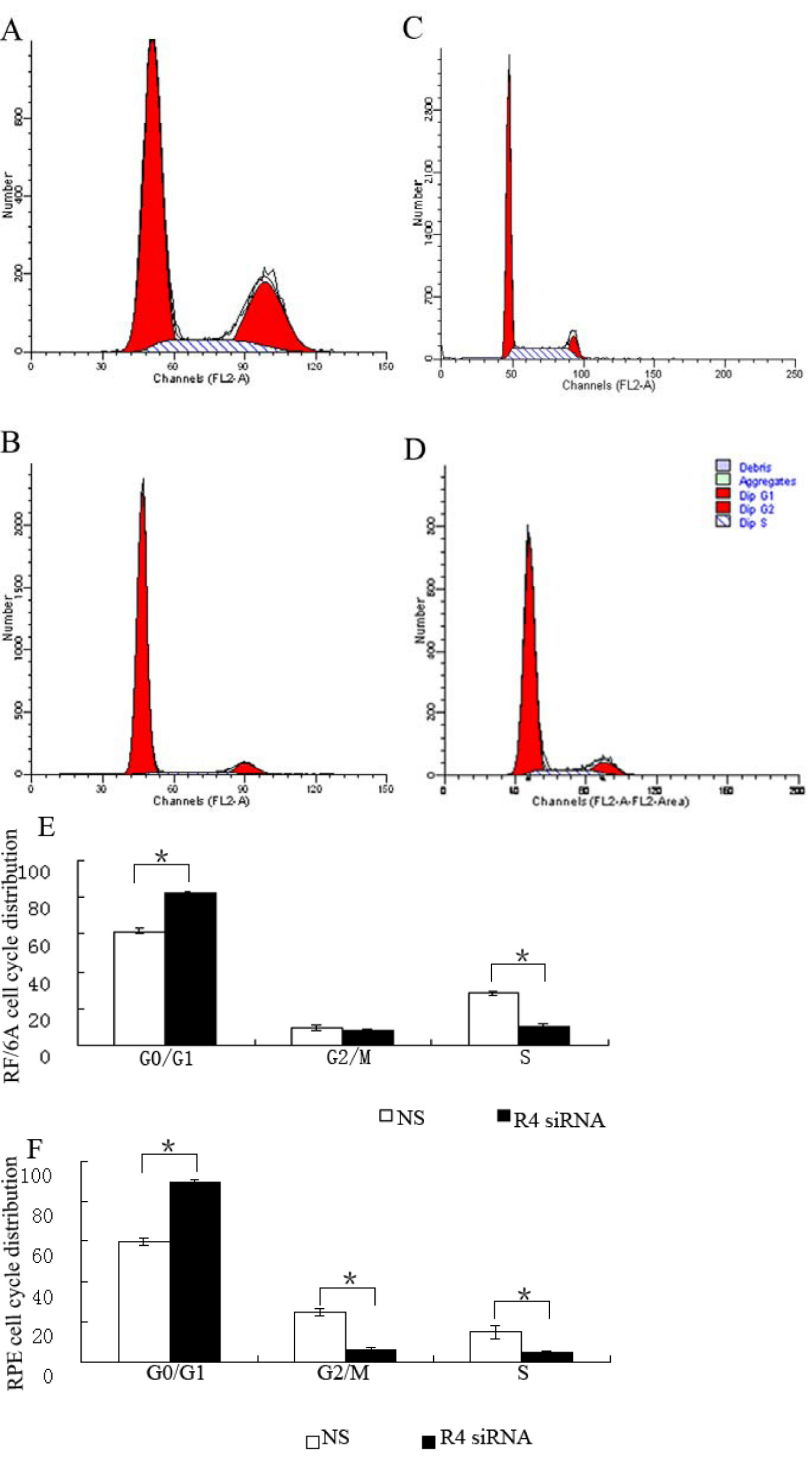
Effect of Robo4 on the cell cycles of RF/6A and human RPE cells. **A:** Cell cycle of NS siRNA-treated RPE cells. **B:** Cell cycle of *Robo4* siRNA-treated RPE cells. **C:** Cell cycle of NS siRNA-treated RF/6A cells. **D:** Cell cycle of *Robo4* siRNA-treated RF/6A cells. **E:** The data of RPE cell cycle distribution of NS and *Robo4* siRNA group cells. F: was the data of RF/6A cell cycle distribution of NS and *Robo4* siRNA group cells. Flow cytometric analysis demonstrated the effects of *Robo4* on the cell cycle. The x-axis represents the fluorescence intensity on a logarithmic scale and the y-axis represents the number of events. The results show that the fraction of G1-phase cells increased and the proportion of S-phase cells decreased in the RF/6A and RPE cells after knockdown of *Robo4*. Values are the means±SD of at least three independent experiments. The proportion of G0/G1, G2, and S phase cells was decreased in *Robo4* siRNA-treated RPE cells compared to NS siRNA-treated RPE cells (**E**, *p<0.01). The proportion of G0/G1 and S phase cells was decreased in *Robo4* siRNA-treated RF/6A cells compared to NS siRNA-treated RF/6A cells (**F**, *p<0.01). Abbreviations: control siRNA-treated cells (NS); *Robo4* siRNA-treated cells (R4 siRNA). The cell cycle of NS was set to 100%.

The depletion of Robo4 levels caused a significant accumulation of cells in G0/G1 phase and a marked reduction in the accumulation of cells in S phase compared with the NS group. Of the *Robo4*-siRNA-treated RPE and RF/6A cells, 89.54% and 81.99% were in G_1_ phase, compared with only 60.17% and 61.91% of the RPE and RF/6A cells, respectively, in the NS group (p<0.01; Figure 12). Of the *Robo4*-siRNA-treated RPE and RF/6A cells, 4.56% and 10.08%, respectively, were in S phase of the cell cycle, compared with 14.98% and 28.47% of the RPE and RF/6A cells, respectively, in the NS group (p<0.01). A slight difference between the RPE and RF/6A cells was observed in G_2_/M phase: 6.23% of *Robo4*-siRNA-treated RPE cells were in G_2_/M phase versus 24.85% of NS RPE cells (p<0.01); whereas there was no significant difference between *Robo4*-siRNA-treated RF/6A cells and NS RF/6A cells (Figure 12).

## Discussion

There is increasing evidence that the migration and patterning of axons and blood vessels share similar guidance mechanisms. There are several gene families with established roles in axon guidance that also control endothelial cell guidance and the angiogenic sprouting of blood vessels. Semaphorins, neuropilin, ephrins/ephs, notch, and delta, are well known as repulsive guidance cues in the nervous system. However, they were also demonstrated to regulate blood vessel branching [22,24,25]. Previous studies of Robo4 have been performed predominantly in zebrafish and mice [7,9] and until now, there has been no report of Robo4 expression in human eye tissue. In this study, we demonstrated for the first time that *Robo4* mRNA is expressed in the FVMs of human eyes with proliferative diabetic retinopathy. The results of dual-color immunofluorecence analysis of the FVMs also showed positive Robo4 staining in the fibrous-like tissue and the neovascular endothelial cells. The Robo4 staining partially colocalized with GFAP, which suggests that Robo4 expression may not be confined to the vessels but also in neural tissues. These observations suggest that Robo4 plays a role in the formation of FVMs.

RNA interference mediated by siRNA is a powerful technology, allowing the silencing of mammalian genes with great specificity and potency. However, nonspecific effects at both the mRNA and protein levels are known to occur with siRNA methods and constitute one of the limitations of this technology [18]. In our study, we observed a significant difference between the *Robo4*-specific siRNA-treated group and the control siRNA-treated group, but there were no differences between the NS siRNA-treated, HF-treated, and untreated controls in all assays. These results suggest that the application of siRNA was experimentally valid and that its transfection induced no cytotoxicity. In our study, *Robo4*-specific siRNA was an effective and specific inhibitor of the attachment, spreading, migration, and proliferation of both RF/6A and RPE cells.

Tube formation is one of the main characteristics of retinal and choroid vascular endothelial cells. Tube formation on Matrigel was used to measure the physiologic effects of Robo4 on RF/6A cells. Robo4-knockdown RF/6A cells showed markedly reduced tubule formation and failed to develop vascular networks on Matrigel. Therefore, Robo4 might play a role in maintaining the tube-forming properties of RF/6A cells.

Cell migration is another principal indicator of the normal physiologic functions of endothelial and epithelial cells. RF/6A and RPE cells transfected with *Robo4* siRNA presented significantly reduced cell migration compared with that of control cells, whereas overexpressed Robo4 in endothelial cells blocked the migration of endothelial cells toward VEGF and fibroblast growth factor [16]. This suggests that the overexpression of *Robo4* resulted in a similar phenotype as that induced by *Robo4* siRNA, implying that too little or too much Robo4 has the same detrimental effect on cell migration [17], and that appropriate *Robo4* expression may sustain the migration properties of cells.

In this study, we investigated whether Robo4 affects the proliferation of RF/6A and RPE cells. No inhibition was apparent in the *Robo4*-siRNA-treated cells for 48 h. The effect on cell growth was observed in both RF/6A and RPE cells and is probably attributable to G_1_/S cell-cycle arrest. The role of Robo4 on the two cell lines was a little different at G_2_/M phase: *Robo4* siRNA reduced the numbers of RPE cells in G_2_/M phase but had no effect on the RF/6A cells. This difference between the two cell types may be attributable to species or cell-type differences: RF/6A is an endothelial cell line, and RPE is an epithelial cell line.

In this study, we exposed RF/6A and RPE cells to hypoxic conditions in vitro to mimic the hypoxia experienced by endothelial and epithelial cells in ischemic retinal and choroid diseases in vivo. We found that under normoxic conditions, there was no significant difference in cell apoptosis between the *Robo4*-siRNA-treated and NS groups, whereas under hypoxic conditions, more apoptotic cells were detected in the *Robo4*-siRNA-treated group than in the NS group. These results suggest that Robo4 does not affect cell apoptosis under normal conditions but increases cell tolerance of hypoxic conditions. This is consistent with a previous study in which a model of retinopathy of prematurity in Robo4AP/AP mice showed increased angiogenesis and vascular leakage [10].

In summary, our study indicates that Robo4 may play a role in the formation of FVMs. Silencing Robo4 expression in RF/6A and RPE cells inhibited their proliferation, migration, spreading, tube formation, and tolerance of hypoxia, and thus may be involved in retina vasculogenesis and angiogenesis.
